# Ginsenoside Rh4 inhibits inflammation-related hepatocellular carcinoma progression by targeting HDAC4/IL-6/STAT3 signaling

**DOI:** 10.1007/s00438-023-02070-w

**Published:** 2023-10-16

**Authors:** Ruiyuan Jiang, Shujuan Luo, Meng Zhang, Wei Wang, Shaoyuan Zhuo, Yajing Wu, Qingmei Qiu, Yuan Yuan, Xiao Jiang

**Affiliations:** 1 Department of Graduate Student, Zhejiang University of Chinese Medicine, Hangzhou, 310000 Zhejiang China; 2 Institute of Basic Medicine and Cancer (IBMC), The Cancer Hospital of the, University of Chinese Academy of Sciences (Zhejiang Cancer Hospital), Hangzhou, 310000 Zhejiang China; 3 Department of Basic Medical Sciences, Faculty of Chinese Medicine Science, Guangxi University of Traditional Chinese Medicine, Nanning, 530022 Guangxi China; 4 Department of Basic Medical Sciences, Guangxi University of Chinese Medicine, No. 13, Wuhe Road, Qingxiu District, Nanning, 530022 Guangxi China; 5 Department of Public Health and Management, Guangxi University of Chinese Medicine, No. 13, Wuhe Road, Qingxiu District, Nanning, 530022 Guangxi China

**Keywords:** Ginsenoside Rh4, Migration, HDAC4, Glycolysis, Hepatocellular carcinoma

## Abstract

This study aimed to investigate the effects of Ginsenoside Rh4 (Rh4) on inflammation-related hepatocellular carcinoma (HCC) progression and the underlying mechanism. HCC cells (HUH7 and LM3) were induced by lipopolysaccharide (LPS) to establish an inflammatory environment in the absence or presence of Rh4. CCK-8, wound healing and transwell assays were employed to analyze the viability, migration and invasion of HCC cells. Ki67 expression was detected by immunofluorescence method. Besides, the levels of glucose and lactic acid were tested by kits. The expression of proteins related to migration, glycolysis and histone deacetylase 4 (HDAC4)/IL-6/STAT3 signaling was measured with western blot. The transplantation tumor model of HCC in mice was established to observe the impacts of Rh4 on the tumor growth. Results indicated that Rh4 restricted the viability and Ki67 expression in HCC cells exposed to LPS. The elevated migration and invasion of HCC cells triggered by LPS were reduced by Rh4. Additionally, Rh4 treatment remarkably decreased the contents of glucose and lactic acid and downregulated LDHA and GLUT1 expression. The database predicated that Rh4 could target HDAC4, and our results revealed that Rh4 downregulated HDAC4, IL-6 and p-STAT3 expression. Furthermore, the enforced HDAC4 expression alleviated the effects of Rh4 on the proliferation, migration, invasion and glycolysis of HCC cells stimulated by LPS. Taken together, Rh4 could suppress inflammation-related HCC progression by targeting HDAC4/IL-6/STAT3 signaling. These findings clarify a new anti-cancer mechanism of Rh4 on HCC and provide a promising agent to limit HCC development.

## Introduction

Primary liver cancer is the sixth most commonly diagnosed cancer and the third leading cause of cancer death worldwide in 2020, with approximately 906,000 new cases and 830,000 deaths (Sung et al. 2021). Hepatocellular carcinoma (HCC) accounts for 70–85% of primary liver cancers and has poor prognosis and high invasiveness (Ghouri et al. 2017; Bray et al. 2018; Villanueva 2019). Early diagnosis of HCC is very challenging, so most patients are diagnosed with advanced stage, when treatment options are limited and ineffective (Brown et al. 2023). Although great progress has been made in the diagnosis and therapies for HCC, the treatment effect of patients with HCC is still unsatisfactory with the overall 5-year survival rate of 25–39%, and the recurrence rate of advanced HCC is about 80% (Llovet et al. 2021). Therefore, an in-depth understanding of the potential mechanism involved in the occurrence and development of HCC is of crucial importance to the development of new therapeutic strategies.

Tumor-promoting inflammation and avoidance of the immune system functions have been reported as novel hallmarks of cancers (Nigam et al. 2023). Chronic inflammation can create a favorable tumor microenvironment, which is conducive to tumor growth and metastasis, and it plays a crucial role in the carcinogenesis of organs (Grivennikov et al. 2010). In the study of liver cancer microenvironment, it has been found that an important feature of liver tumor microenvironment is the persistence of bacterial antigen components, such as lipopolysaccharide (LPS) (Gu et al. 2015). Previous studies have shown that LPS can induce the proliferation, invasion and migration of HCC cells (Lu et al. 2018; Liu et al. 2021). Therefore, identification of LPS-induced endogenous inflammatory molecules and effective pharmacological intervention may provide the possibility for the development of effective treatment methods for HCC.

Ginsenosides are the main active components of ginseng, a rare perennial herb widely used in traditional and modern medicine, which are responsible for the biological and pharmacological activities, such as antitumor, anti-inflammatory and immune enhancement effects (Gao et al. 2022; Yao and Guan 2022; Zhang et al. 2022a). Ginsenoside Rh4 (Rh4), a kind of tetracyclic triterpene saponins, is known to be endowed with an inhibitory effect on the development of diverse cancers (Huang et al. 2023). For instance, Rh4 exhibits the anti-metastatic effect on the lung adenocarcinoma and gastric cancer by restraining the proliferation, migration and invasion of cancer cells (Jiang et al. 2022; Zhang et al. 2022b). By inhibiting aerobic glycolysis, Rh4 plays anti-esophageal cancer roles with few side effects (Deng et al. 2020). Besides, the low toxic side effects and anti-inflammatory effects of the Rh4 have also attracted extensive attention from researchers (Bai et al. 2021; To et al. 2022). However, the role of Rh4 in LPS-induced aggressive progression of HCC cells has not been reported.

This study was performed to investigate the impacts of Rh4 on the regulation of proliferation, migration, invasion and glycolysis of HCC cell lines (HUH7 and LM3) and transplantation tumor model of HCC in mice. Further, the potential mechanisms related to histone deacetylase 4 (HDAC4)/ interleukin (IL)-6/signal transducer and activator of transcription 3 (STAT3) signaling were explored.

## Materials and methods

### Cell culture and treatment

Human HCC cell lines HUH7 and LM3 cells procured from Cell Bank of Shanghai Institutes of Biological Sciences, Chinese Academy of Sciences (Shanghai, China) were cultivated in Dulbecco’s modified Eagle’s medium (DMEM; Lonza, Basal, Switzerland) containing 10% fetal bovine serum (FBS; Gibco, LifeTech, USA) in a humidified incubator with 37 °C and 5% CO_2_. Cells were cultured 48 h after treatment with 1 µg/ml LPS (Sigma-Aldrich; Merck KGaA, Darmstadt, Germany) in the presence or absence of pretreatment with different concentrations of Rh4 (40, 60 and 80 µM) for 30 min (Dong et al. 2015; Deng et al. 2020; Wang et al. 2021). Rh4 (purity ≥ 99%) was purchased from Puruifa Technology Development Co., Ltd. (Chengdu, China).

### Cell transfection

For transfection, the HDAC4 overexpression plasmid (Ov-HDAC4) and the empty vector plasmid (Ov-NC) were synthesized by GenePharma (Shanghai, China). HUH7 and LM3 cells at the logarithmic phase were seeded in a 6-well plate with the density of 1 × 10^5^ cells per well and incubated at 37 °C. Cell transfection was performed at 80% confluence with the application of Lipofectamine 2000 (Invitrogen) according to the manufacture’s protocol. Following transfection for 48 h, the transfected cells were induced by Rh4 and LPS as described above.

### Cell viability assay

Cell viability was assessed using the (CCK-8) kit obtained from Beyotime (Shanghai, China). After transfection and the indicated treatment with LPS and Rh4, cells were incubated with CCK-8 solution for 2 h at 37 °C. The absorbance was recorded at 450 nm by using a microplate reader (Rayto, Ltd., China).

### Immunofluorescence staining

After the indicated treatment, HUH7 and LM3 cells were fixed with 4% paraformaldehyde for 15 min at 37 °C, followed by permeabilization with 0.1% Triton X-100 for 20 min. After blocking with 4% bovine serum albumin (BSA), cells were incubated with primary antibody against Ki67 (cat. no. ab16667; Abcam Company, Cambridge, UK) overnight at 4 °C. PBS was used to wash the slides for three times, and then secondary antibody was added for 1 h incubation at room temperature. The cell nuclei were stained by DAPI. Cells was observed and imaged by an inverted fluorescence microscope (Olympus, Tokyo, Japan).

### Wound healing assay

HUH7 and LM3 cells were inoculated into six-well plates at the density of 1 × 10^5^ cells/well. A pipette was used to make a straight line in the center of the plate. After rinsed with PBS buffer, the cells were cultured in a serum-free medium for 48 h. The width of the scratch was observed with a microscope and measured by ImageJ software (National Institutes of Health, Bethesda, MD, USA).

### Transwell assay

Cell invasion assay was performed using transwell chambers which were coated with Matrigel (BD Biosciences, USA). Following transfection, HUH7 and LM3 cells were collected and 5 × 10^4^ cells were added to the 200 μl serum-free medium in the upper chambers. The bottom chamber was supplemented with 800 μl DMEM containing 10% FBS as chemo-attractant. After incubation for 48 h, the cells in the upper chamber were removed and the obtained cells were fixed with paraformaldehyde and stained with 0.1% crystal violet for 10 min. The invasive cells were observed by an inverted microscope (Olympus, Tokyo, Japan).

### Detection of glucose and lactic acid

Following the indicated treatment, HUH7 and LM3 cells were collected and lysed in RIPA lysis buffer (Beyotime; Shanghai, China), followed by centrifugation to obtain the supernatant. The levels of glucose and lactic acid were detected with the application of the corresponding assay kits provided by Nanjing Jiancheng Bioengineering Institute (Nanjing, China).

### Human HCC xenograft nude mouse model

The total 15 male BALB/c nude mice (5 weeks old, weighing about 21 g) were purchased from GemPharmatech (Jiangsu, China). The tumors were xenografted into the left flank of nude mice through subcutaneous injection of 2 × 10^6^ cells in 50 μl of PBS. The mice were intratumorally injected with LPS (400 μg/kg) or the same volume of DMSO every other day. The tumor growth in the different groups was observed every two days. After 3 weeks, all mice were sacrificed, and the tumors were dissected. Tumor size was measured and calculated using the equation (length × width^2^)/2. All animal studies were approved by the Animal Ethics Committee of Guangxi University of Chinese Medicine and experiments were conducted according to the Animal Management Rules of the Chinese Ministry of Health.

### Molecular docking

The 3D structure of HDAC4 was downloaded from the PDB website (http://www.rcsb.org/) and saved in PDB format. The files were converted by AutoDockTools 1.5.6 into the “pdbqt” format. Finally, Autodock (version 4.2) was used for molecular docking, and the results were visualized using Pymol 2.5.2 (https://pymol.org/2/).

### Western blot

Proteins in cells and tumor tissues were lysed with RIPA lysis buffer (Beyotime; Shanghai, China). A BCA protein assay kit was used to detect protein concentrations (Beyotime; Shanghai, China). Protein samples were loaded onto 10% SDS-PAGE and transferred to a polyvinylidene fluoride (PVDF) membrane (Merck Millipore, Darmstadt, Germany). After blocking in 5% nonfat milk for 1 h, the protein bands were incubated overnight with primary antibodies at 4 °C. On the second day, the second antibodies (cat. no. 7074P2; 1:5000; Cell Signaling Technology, Boston, MA, USA) were added for 1 h of incubation and the membranes were then developed with an enhanced chemiluminescence kit (Thermo Fisher Scientific). The band intensities were quantified using ImageJ software with respect to β-actin. Anti- matrix metalloproteinases 2 (MMP2; cat. no. 87809S; 1:1000), anti-lactate dehydrogenase A (LDHA; cat. no. 3582T; 1:1000), anti-glucose transporter type 1 (GLUT1; cat. no. 73015S; 1:1000), anti-HDAC4 (cat. no. 7628T; 1:1000), anti-photo (p)-STAT3 (cat. no. 9145T; 1:1000), anti-STAT3 (cat. no. 4904T; 1:1000) and anti-β-actin (cat. no. 4970T; 1:1000) antibodies were provided by Cell Signaling Technology (Boston, MA, USA). Anti-MMP9 (cat. no. ab283575; 1:1000), anti-IL-6 (cat. no. ab259341; 1:1000) and anti-Ki67 (cat. no. ab16667) antibodies were obtained from Abcam Company (Cambridge, UK).

### Statistical analysis

Data were presented as mean ± standard deviation (SD). All experiments were carried out at least three times and presented with representative data. The statistical graph was generated by GraphPad 8.0 statistical software (GraphPad Software Inc., USA). Statistical calculations of the data were performed using one-way analysis of variance (ANOVA), followed by Tukey’s test. A *p*-value less than 0.05 was considered a significant difference.

## Results

### Rh4 treatment inhibits the proliferation, migration and invasion of LPS-induced HCC cells

**Fig. 1:**
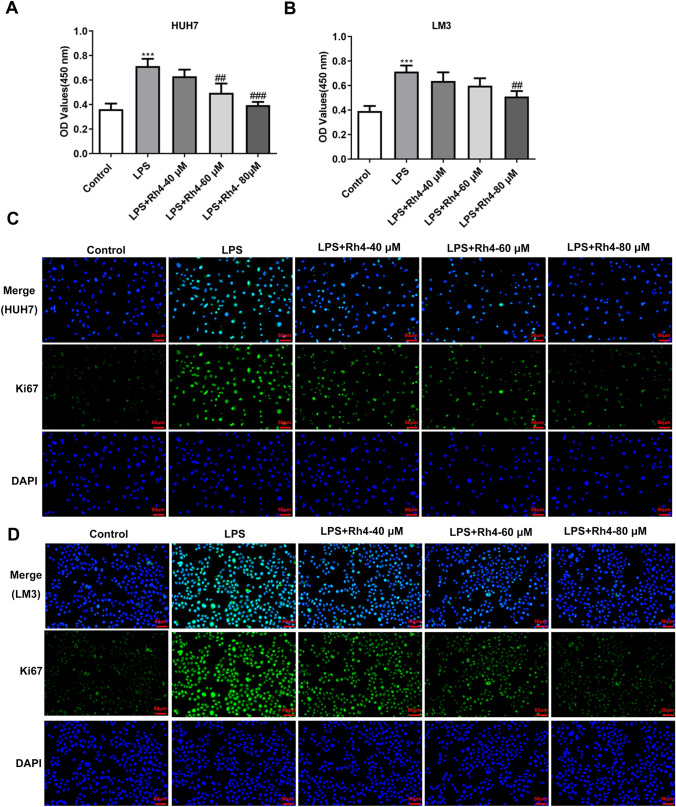
Rh4 treatment inhibited the proliferation of LPS-induced HCC cells. The viability of **A** HUH7 and **B** LM3 cells stimulated by LPS in the presence or absence of Rh4 was evaluated by CCK-8 assay. Ki67 expression in **C** HUH7 and **D** LM3 cells treated by LPS or/and Rh4 was tested by immunofluorescence staining. Magnification, × 200. ^***^P < 0.001 vs. control group; ^##^*P* < 0.01, ^###^*P* < 0.001 vs. LPS group

**Fig. 2:**
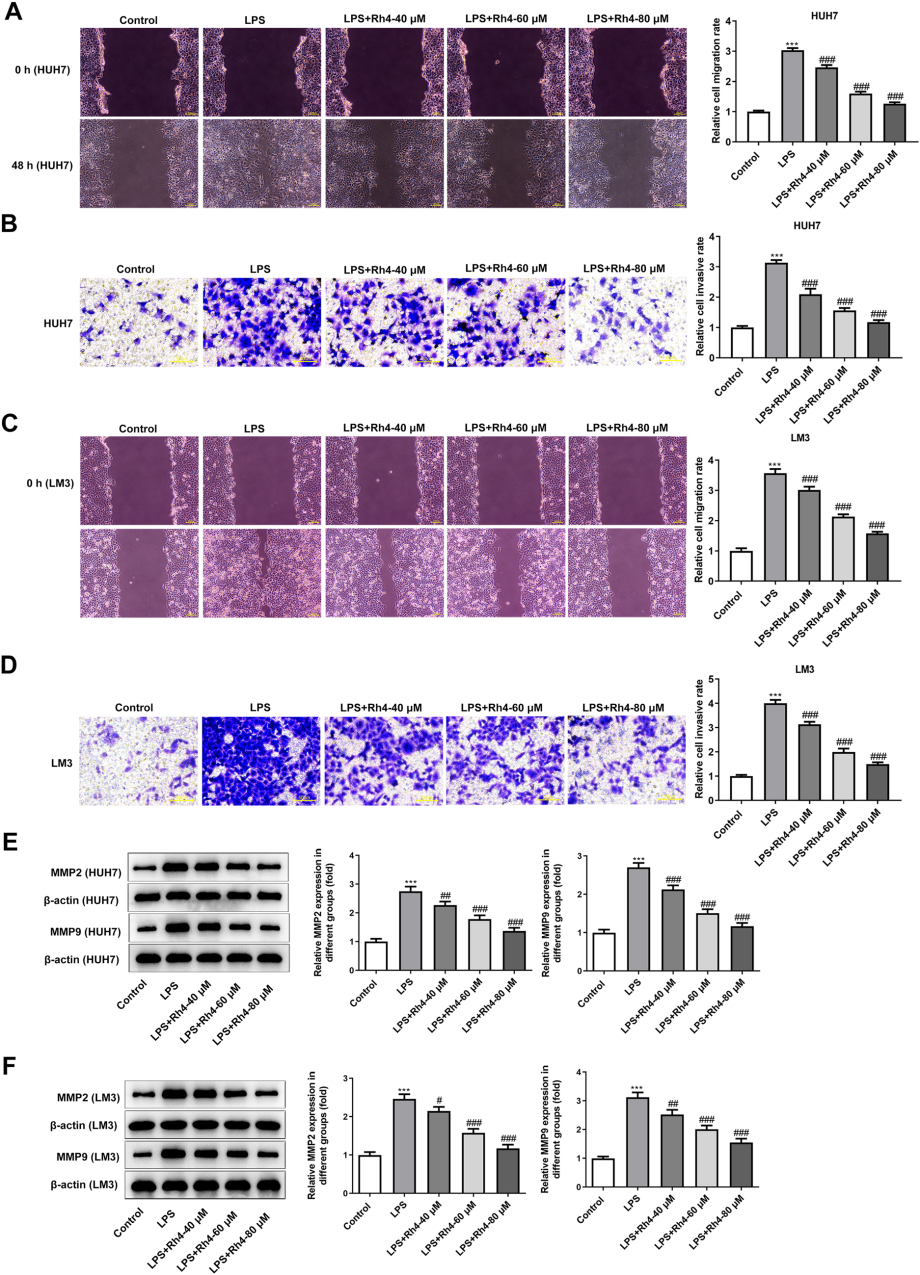
Rh4 treatment inhibited the migration and invasion of LPS-induced HCC cells. The capacities of **A** migration and **B** invasion of HUH7 cells were measured with the help of wound healing and transwell assay. The capacities of **C** migration and **D** invasion of LM3 cells were determined using wound healing and transwell assay. Magnification, wound healing, × 100, transwell, × 200. The expression of MMP2 and MMP9 proteins in **E** HUH7 and **F** LM3 cells was estimated by western blot assay. ^***^P < 0.001 vs. control group; ^#^*P* < 0.05, ^##^*P* < 0.01, ^###^*P* < 0.001 vs. LPS group

It has been reported that LPS can induce the malignant phenotypes of HCC cells (Lu et al. 2018; Liu et al. 2021). To investigate the effects of Rh4 on LPS-induced increase in cell proliferation, migration and invasion, two types of HCC cells were subjected to treatment with LPS and Rh4. Firstly, the viability of LPS-induced HUH7 and LM3 cells after Rh4 administration was evaluated by means of CCK-8 assay. As depicted in Fig. 1A, B, LPS stimulation obviously elevated the viability of both HUH7 and LM3 cells when compared to the control group. On the contrary, the addition of Rh4 dose-dependently reduced the viability of these two HCC cell lines. Consistently, the enhanced fluorescence intensities of Ki67 induced by LPS in HUH7 and LM3 cells were significantly decreased with the increase of Rh4 concentrations (Fig. 1C, D). Additionally, Rh4 remarkably inhibited the migration (Fig. 2A) and invasion (Fig. 2B) of HUH7 cells exposed to LPS. As exhibited in Fig. 2C, D, the same trend of migrative and invasive capacities in the presence or absence of LPS and Rh4 was also found in LM3 cells. Concurrently, LPS led to upregulated MMP2 and MMP9 expression in both HUH7 and LM3 cells compared to the control group, which were gradually restored by the increase in the concentration of Rh4 (Fig. 2E, F). These data suggest that Rh4 treatment suppresses the proliferation, migration and invasion of LPS-induced HCC cells.

### Rh4 treatment suppresses the glycolysis of LPS-induced HCC cells

**Fig. 3:**
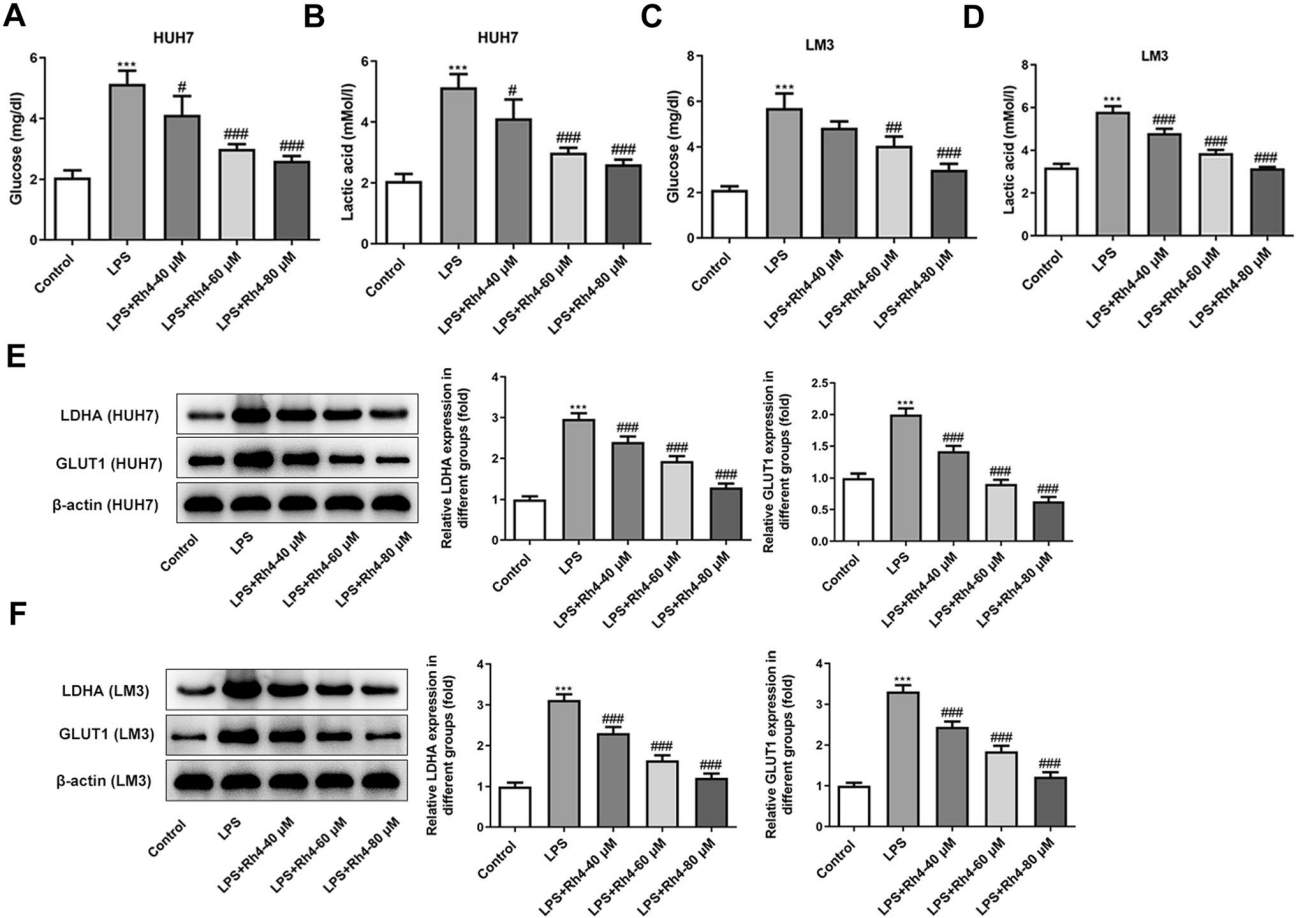
Rh4 treatment suppressed the glycolysis of LPS-induced HCC cells. The levels of **A** glucose and **B** lactic acid in HUH7 cells were detected by the corresponding kits. The levels of **C** glucose and **D** lactic acid in LM3 cells were estimated with the application of the corresponding kits. Western blot was employed to analyze the expression of LDHA and GLUT1 in **E** HUH7 and **F** LM3 cells. ^***^P < 0.001 vs. control group; ^#^*P* < 0.05, ^##^*P* < 0.01, ^###^*P* < 0.001 vs. LPS group

This section was conducted to analyze the impacts of Rh4 on the autophagy and glycolysis in HCC cells. The glycolysis levels of LPS-induced HCC cells were evaluated in HUH7 and LM3 cells when treated with LPS and Rh4. It was found that LPS exposure resulted in obviously elevated glucose (Fig. 3A) and lactic acid (Fig. 3B) contents in HUH7 cells relative to the control group. When compared to the LPS group, the levels of glucose and lactic acid were dose-dependently decreased after the addition of Rh4. Meantime, LM3 cells stimulated with LPS and Rh4 showed the same variation tendency on glucose and lactic acid as HUH7 cells (Fig. 3C, D). LDHA and GLUT1 are two key regulatory enzymes involving in glycolysis in the development of HCC (Ye et al. 2019). Results of western blot exhibited in Fig. 3E, F indicated that LPS-induced upregulation in LDHA and GLUT1 expression in both HUH7 and LM3 cells was downregulated by Rh4 treatment and the most obvious inhibitory effect was observed in the Rh4-80 μM group. These observations reveal that Rh4 treatment retrains the glycolysis of HCC cells under LPS stimulation.

### Rh4 can target HDAC4 to inactivate the HDAC4/IL-6/STAT3 signaling in HCC cells

**Fig. 4:**
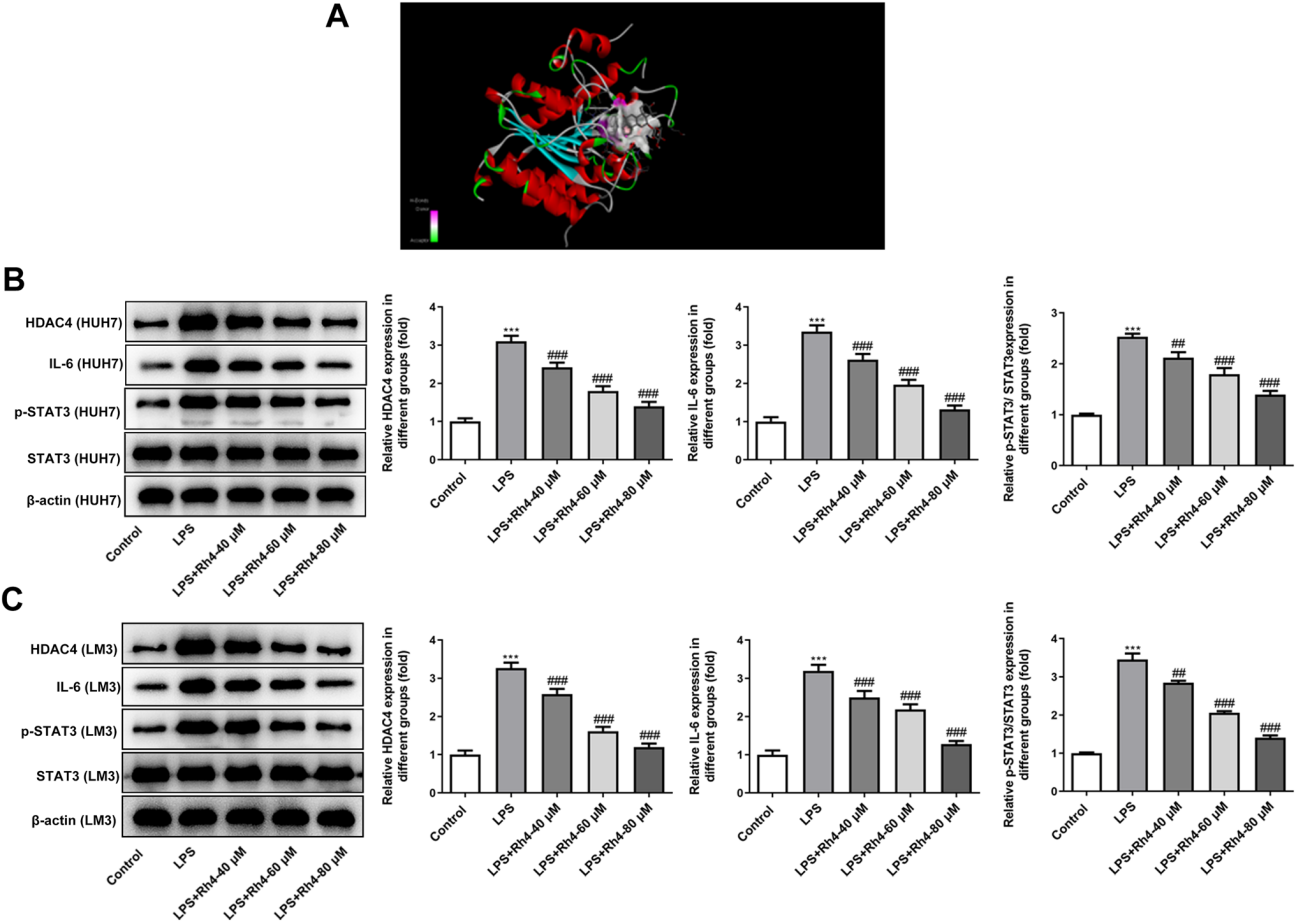
Rh4 targeted HDAC4 to inactivate the HDAC4/IL-6/STAT3 signaling in HCC cells. **A** Stereo view of the binding mode for Rh4 with HDAC4. **B** The expression of proteins in HDAC4/IL-6/STAT3 signaling in HUH7 cells was tested using western blot assay. **C** The expression of proteins in HDAC4/IL-6/STAT3 signaling in LM3 cells was tested using western blot assay. ^***^P < 0.001 vs. control group; ^##^*P* < 0.01, ^###^*P* < 0.001 vs. LPS group

To explore the mechanisms of Rh4 in regulating the malignant biological behaviors of HCC cells, molecular docking method was used to predict the protein that could be targeted by Rh4, and it was found that HDAC4 served as a potential protein that bound to Rh4 (Fig. 4A). Additionally, western blot demonstrated that LPS-induced increase in HDAC4 expression levels in both HUH7 and LM3 cells were reduced by Rh4 in a dose-dependent manner (Fig. 4B, C). To further investigate the mechanism underlying the effects of Rh4 on the LPS-induced enhancement of malignant behaviors in HCC cells, we detected the expression of proteins in IL-6/STAT3 signaling. It was found that Rh4 significantly downregulated the expression of IL-6 and p-STAT3 in LPS-induced HUH7 and LM3 cells as comparison to the LPS group. Above data reveal that Rh4 targets HDAC4 to inactivate the HDAC4/IL-6/STAT3 signaling in HCC cells.

### HDAC4 overexpression weaken the impacts of Rh4 on the progression of HCC cells

**Fig. 5:**
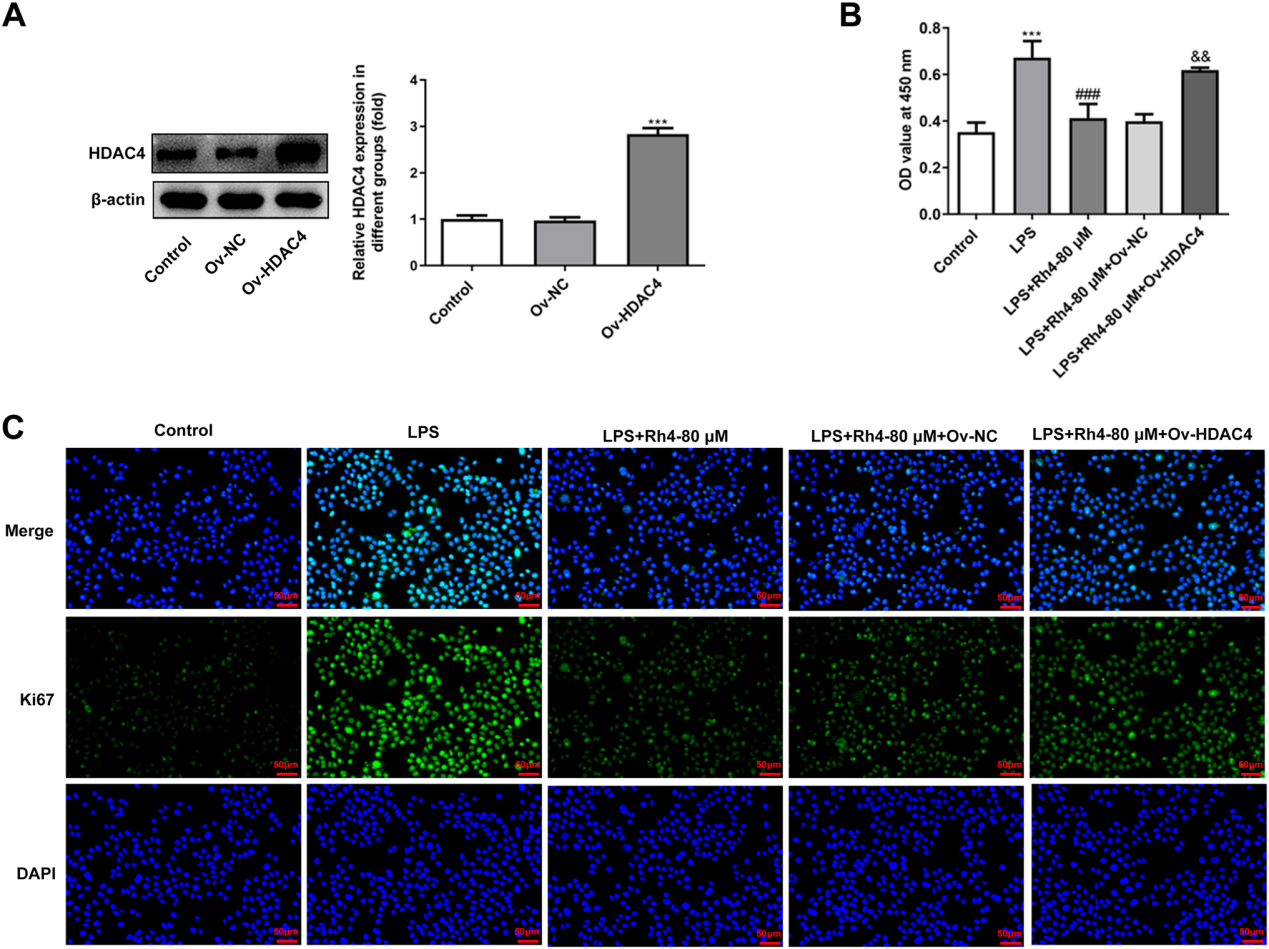
HDAC4 overexpression alleviated the impacts of Rh4 on the proliferation of HUH7 cells treated with LPS and Rh4. **A** HDAC4 level in HUH7 cells was tested by western blot after transfection. ^***^P < 0.001 vs. Ov-NC group. **B** Cell viability was detected by CCK-8 assay. ^***^P < 0.001 vs. control group; ^###^*P* < 0.001 vs. LPS group; ^&&^*P* < 0.01 vs. LPS + Rh4-80 μM + Ov-NC group. **C** Ki67 expression was analyzed by immunofluorescence staining. Magnification, × 200

**Fig. 6:**
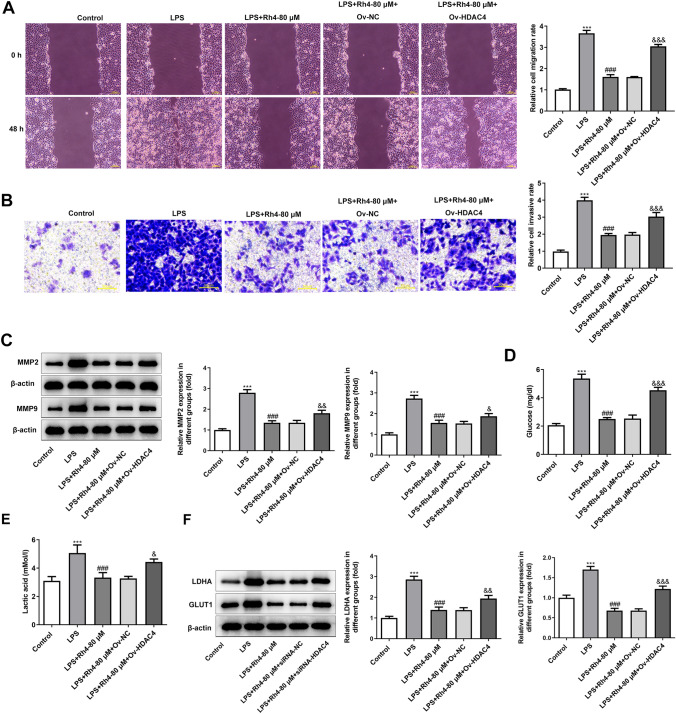
HDAC4 overexpression attenuated the impacts of Rh4 on the migration, invasion and glycolysis of HUH7 cells treated with LPS and Rh4. The capacities of **A** migration and **B** invasion of HUH7 cells were measured with the help of wound healing and transwell assay. Magnification, wound healing, × 100, transwell, × 200. **C** The expression of MMP2 and MMP9 proteins was estimated by western blot assay. The levels of **D** glucose and **E** lactic acid in HUH7 cells were detected by the corresponding kits. **F** Western blot was employed to analyze the expression of LDHA and GLUT1. ^***^P < 0.001 vs. control group; ^###^*P* < 0.001 vs. LPS group; ^&^*P* < 0.05, ^&&^*P* < 0.01, ^&&&^*P* < 0.001 vs. LPS + Rh4-80 μM + Ov-NC group

Due to the better inhibitory effects, Rh4-80 μM was selected to treat HUH7 cells in the following experiments. And HDAC4 was overexpressed to clarify the regulatory effects of Rh4 on HDAC4/IL-6/STAT3 signaling in HCC cells. Significantly elevated HDAC4 expression was observed in HUH7 cells transfected with HDAC4 plasmid (Fig. 5A). CCK-8 assay and Ki67 staining indicated that HDAC4 gain-of-function partially reversed the decreased HUH7 cell viability and Ki67 fluorescence intensity caused by Rh4-80 μM administration under LPS stimulation (Fig. 5B, C). As expected, the migration and invasion of HUH7 cells were obviously promoted after transfection with HDAC4 plasmid in the presence of LPS and Rh4 (Fig. 6A, C). Besides, it was observed from western blot assay that HDAC4 overexpression markedly upregulated MMP2 and MMP9 expression in HUH7 cells when compared to the LPS + Rh4-80 μM + Ov-NC group (Fig. 6C). The further experiments revealed that the decrease in glucose and lactic acid contents as well as LDHA and GLUT1 expression levels caused by Rh4 was restored after HDAC4 was overexpressed (Fig. 6D, E). Through the above findings, we prove that HDAC4 overexpression weaken the impacts of Rh4 on the progression of HCC cells.

### Rh4 inhibits the development of subcutaneous HUH7 tumors in nude mice and inactivates the HDAC4/IL-6/STAT3 signaling

**Fig. 7:**
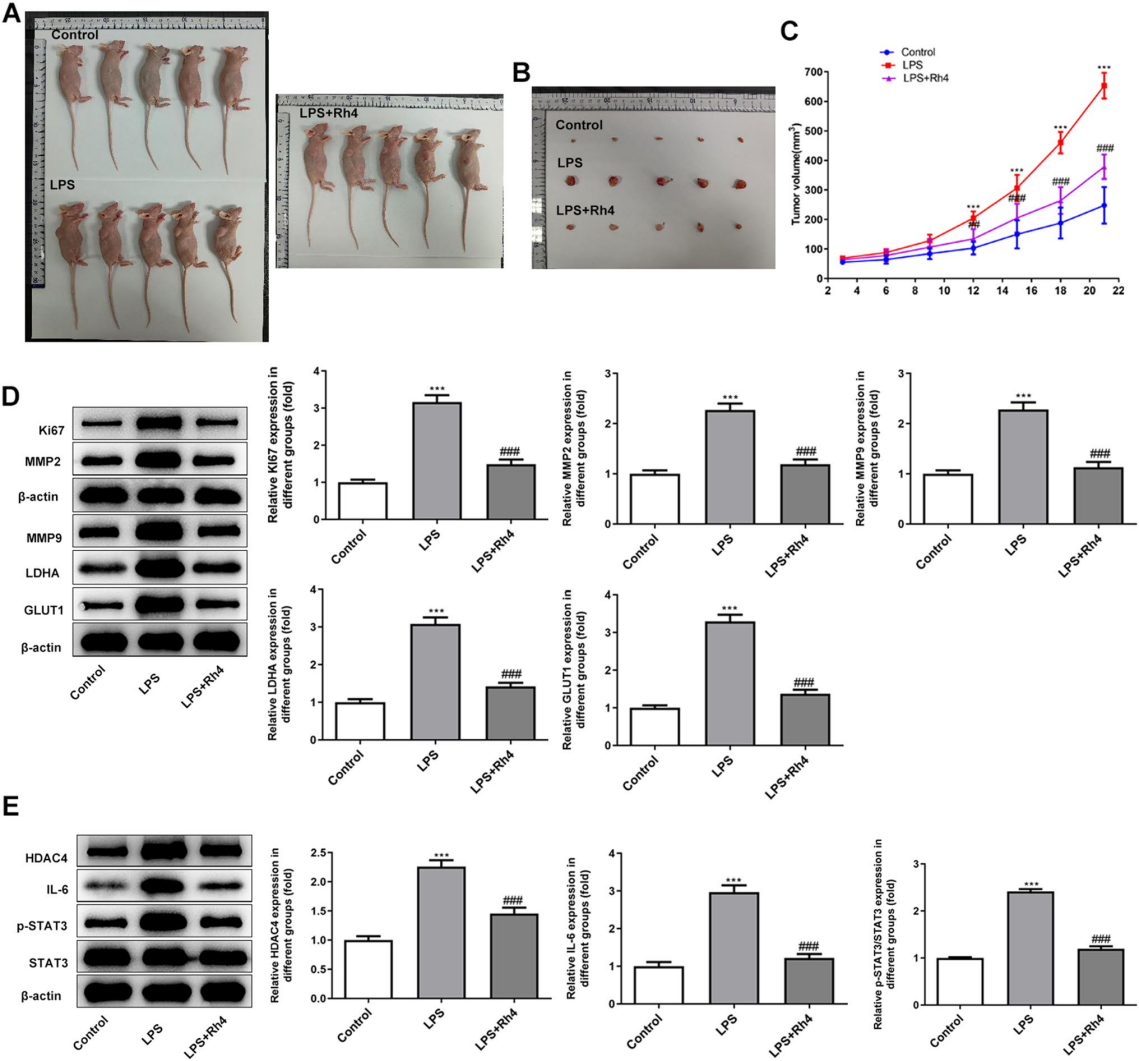
Rh4 repressed the development of subcutaneous HUH7 tumors in nude mice and inactivates the HDAC4/IL-6/STAT3 signaling. **A** Representative images of HUH7 xenograft nude mice in the control group, LPS group and LPS + Rh4 group. **B** Representative images of HUH7 xenograft tumors of the control group, LPS group and LPS + Rh4 group. **C** The tumor volume was recorded every two days. **D** The expression of Ki67, MMP2, MMP9, LDHA and GLUT1 in tumor tissues of nude mice was detected with the application of western blot. **E** The expression of HDAC4, IL-6 and p-STAT3 in tumor tissues of nude mice was detected with the application of western blot. ^***^P < 0.001 vs. control group; ^##^*P* < 0.01, ^###^*P* < 0.001 vs. LPS group

Then, the transplantation tumor model of HUH7 cells in mice was established to analyze the antineoplastic effect of Rh4 in vivo. As shown in Fig. 7A–C, LPS stimulation notably increased the tumor volume of nude mice compared with the control group, which was decreased after Rh4 administration. In addition, the upregulation in Ki67, MMP2, MMP9, LDHA and GLUT1 expression caused by LPS was also downregulated by Rh4 treatment (Fig. 7D). Consistently, Rh4 reduced the expression levels of HDAC4, IL-6 and p-STAT3 in tumor tissues of nude mice intratumorally injected with LPS as comparison to the LPS group (Fig. 7E). In general, these observations reveal that Rh4 represses the growth of subcutaneous HUH7 tumors in nude mice and inactivates the HDAC4/IL-6/STAT3 signaling.

## Discussion

The association between chronic inflammation and the development and metastatic progression of human cancers has been extensively studied. Chronic inflammation of the liver is a well-recognized risk factor for carcinogenesis since 80% of HCC cases are associated with cirrhosis or fibrosis (Alison et al. 2011; Afify et al. 2022; Wen et al. 2022). In addition, chronic inflammation can promote the malignant development of liver cancer and affect the prognosis (Leone et al. 2021). Therefore, it is of great significance to explore and develop anti-liver cancer drugs from the perspective of inflammation. In this study, we focused on the effects and underlying regulatory mechanism of Rh4 on malignant progression of LPS-induced HCC cells. We finally proved that Rh4 could suppress inflammation-related HCC progression by targeting HDAC4/IL-6/STAT3 signaling.

In recent years, traditional Chinese medicine have made good progress in the treatment of HCC due to their advantages of high efficiency, low toxicity and side effects (Xi and Minuk 2018; Yuan et al. 2023). For instance, Schisantherin A has been reported to inhibit cell proliferation by regulating glucose metabolism pathway in HCC (Feng et al. 2022). Tao et al. have demonstrated that Orientin, a flavone isolated from medicinal plants used in traditional Chinese medicine, represses the proliferation and migration of HCC cells (Tao et al. 2023). From Jean’s work, total flavonoids from Radix Tetrastigma inhibit inflammation-related proliferation and invasion of HCC cells (Liu et al. 2021). Particularly, Ginsenoside Rk3 has excellent efficacy in alleviating intestinal inflammatory response and protecting the liver, and it can inhibit the development of HCC by targeting the gut-liver axis (Qu et al. 2021). As a rare triol ginsenoside, Rh4 is more soluble in water than other polysaccharides ginsenoside, which might improve its prospects for clinical application (Baek et al. 1996). A growing body of literature has shown that Rh4 has excellent anti-inflammatory and anti-cancer properties. Rh4 inhibits LPS-induced activation of NF-κB and STAT3 signaling in macrophages, thereby playing anti-inflammatory effect (To et al. 2022). Rh4 can also protect against ethanol-induced gastric mucosal injury via mitigating inflammation and oxidative stress (Wu et al. 2023). Shao et al.’s research findings have suggested that Rh4 can inhibit the apoptosis of hippocampal neurons and the damage of synaptic structure caused by the overexpression of proinflammatory cytokines and the over-activation of microglia and astrocytes by inhibiting the immune inflammatory response (Shao et al. 2023). Besides, Rh4 has been reported to inhibit the proliferation of colorectal cancer cells (Wu et al. 2022). By regulating immune microenvironment and apoptosis, Rh4 suppresses the growth of breast cancer without any adverse effects (Dong et al. 2023). In a study of esophageal squamous cell carcinoma, Chen's team has found that Rh4 inhibits tumor metastasis both in vitro and in vivo (Chen et al. 2022). This work was the first to reveal the effects of Rh4 on malignant progression of LPS-induced HCC cells, and we demonstrated that Rh4 treatment inhibited the proliferation, migration and invasion of LPS-induced HCC cells.

It is well known that tumor cells under physiological oxygen conditions tend to be metabolized by glycolysis rather than oxidative phosphorylation, exhibiting an abnormally high rate of aerobic glycolysis, termed the Warburg effect (Chandel 2021). Upregulation of aerobic glycolysis is thought to underlie tumor cell survival and tumorigenesis (Chelakkot et al. 2023). There are a plenty of related enzymes participating in the regulation of glycolysis. For instance, LDHA, an isoenzyme of lactate dehydrogenase that functions in the final step of glycolysis to convert pyruvate to lactate, has been demonstrated to be elevated in tumors and can be used as a prognostic marker in cancer patients (Koukourakis and Giatromanolaki 2019). Notably, GLUT1 is a transmembrane protein responsible for the uptake of glucose into the cells in a readily diffused manner (Wang et al. 2019a). Glucose metabolism produces lactic acid, which is the main end product of glucose metabolism (Mossenta et al. 2020). Therefore, the estimation of extracellular lactic acid and glucose may indirectly reflect the metabolic level of aerobic glycolysis (Fang et al. 2019). Recent studies have suggested that glycolysis metabolism is closely associated with the development and progression of HCC (Bi et al. 2021; Li et al. 2022; Lin et al. 2023). A better understanding of the interaction between glycolysis and anti-tumor agents is of great importance to understand the pathogenesis and therapeutic mechanisms of cancers. Importantly, Rh4 plays anti-esophageal cancer roles with few side effects by inhibiting aerobic glycolysis (Deng et al. 2020). In the current study, Rh4 dose-dependently suppressed the glycolysis of both HUH7 and LM3 cells under LPS condition, evidenced by decreased glucose and lactic acid contents as well as downregulated LDHA and GLUT1 expression.

To explore the mechanisms of Rh4 in regulating the malignant biological behaviors of HCC cells, molecular docking method found that HDAC4 served as a potential protein that bound to Rh4. HDAC4, located on chromosome 2q37.2, is a member of class IIa family of HDACs which are hotspots in the field of cancer drug development (Wang et al. 2014). An increasing body of evidence suggests that HDAC4 plays a crucial role in tumorigenesis and is frequently dysregulated in human malignancies (Cuttini et al. 2023; Xu et al. 2023). HDAC4 has been considered as a promising therapeutic target for multiple cancers, including gastric cancer, nasopharyngeal carcinoma and colon cancer, due to its effects on promoting the growth and metastasis of tumors (Cheng et al. 2021; Zang et al. 2022). A previous study has highlighted that HDAC4 expression is increased in the tissues of type B HCC, and the higher the expression, the worse the prognosis (Wang et al. 2019b). Of note, Zhang et al. have also reported that HDAC4 knockdown has an anti-proliferative effect on HCC cells both in vitro and in vivo (Zhang et al. 2010). The results of this work suggested that the upregulated HDAC4 expression induced by LPS was downregulated by Rh4 in a dose-dependent manner. Additionally, IL-6/STAT3 is one of the key signaling involved in the occurrence, development and invasion of HCC cells (Liu et al. 2010, 2022). In recent years, IL-6/STAT3 has been thought to be a promising therapeutic target for HCC (Xu et al. 2021). Importantly, HDAC4-related inhibitors can inhibit the activation of downstream IL-6/STAT3 signaling to suppress the growth and metastasis of breast cancer (Chen et al. 2020). In the present study, Rh4 led to the inactivation of IL-6/STAT3 signaling in LPS-stimulated HCC cells and in the tissues of mouse HCC subcutaneous transplanted tumor, evidenced by reduced IL-6 and p-STAT3 expression. Moreover, HDAC4 overexpression restored the impacts of Rh4 on the malignant phenotypes of HCC cells, suggesting that Rh4 inhibited the development of HCC by inactivating the HDAC4/IL-6/STAT3 signaling. It has been reported that autophagy-mediated ferroptosis plays significant roles in the progression of HCC (Li et al. 2021; Hu et al. 2022). As reported, Rh4 induces autophagic cell death and ferroptosis to inhibit the malignant progression of cancers (Wu et al. 2018; Ying et al. 2023). Therefore, our next experiments will explore whether Rh4 affects autophagy and ferroptosis in HCC.

Taken together, the findings of this study demonstrated that Rh4 inhibited the development of HCC under LPS-induced inflammatory condition both in vitro and in vivo. In detail, Rh4 targeted and suppressed HDAC4/IL-6/STAT3 signaling to exert the anti-HCC effects. These findings might clarify a new anti-cancer mechanism of Rh4 on HCC and provide a promising agent for the treatment of HCC.

## Data Availability

Enquiries about data availability should be directed to the corresponding author.
